# Gene regulation network analyses of pistil development in papaya

**DOI:** 10.1186/s12864-021-08197-7

**Published:** 2022-01-05

**Authors:** Zhenyang Liao, Fei Dong, Juan Liu, Lele Xu, Amy Marshall-Colon, Ray Ming

**Affiliations:** 1 Center for Genomics and Biotechnology, Fujian Provincial Key Laboratory of Haixia Applied Plant Systems Biology, Fujian Agriculture and Forestry University, Fujian 350002 Fuzhou, China; 2 The Wuhan Botanical Garden of the Chinese Academy of Sciences, Wuhan, 430074 Hubei China; 3 Department of Plant Biology, University of Illinois at Urbana-Champaign, Urbana, IL 61801 USA

**Keywords:** *Carica papaya*, Pistil development, Gene regulatory network, Transcription factor

## Abstract

**Background:**

The pistil is an essential part of flowers that functions in the differentiation of the sexes and reproduction in plants. The stigma on the pistil can accept pollen to allow fertilization and seed development. Papaya (*Carica papaya* L.) is a dioecious plant, where female flowers exhibit normal pistil, while the male flowers exhibit aborted pistil at a late stage of pistil development.

**Results:**

The developmental stages of papaya pistil were analyzed after first dividing it into slices representing the primordium stage 1 (S1), the pre-meiotic stages S2, post-meiotic stage S3, and the mitotic stage S4. The SS scoring algorithm analysis of genes preferentially expressed at different stages revealed differentially expressed genes between male and female flowers. A transcription factor regulatory network for each stage based on the genes that are differentially expressed between male and female flowers was constructed. Some transcription factors related to pistil development were revealed based on the analysis of regulatory networks such as *CpAGL11*, *CpHEC2*, and *CpSUPL*. Based on the specific expression of genes, constructed a gene regulatory subnetwork with *CpAGL11-CpSUPL-CpHEC2* functioning as the core. Analysis of the functionally enriched terms in this network reveals several differentially expressed genes related to auxin/ brassinosteroid signal transduction in the plant hormone signal transduction pathway. At the same time, significant differences in the expression of auxin and brassinosteroid synthesis-related genes between male and female flowers at different developmental stages were detected.

**Conclusions:**

The pistil abortion of papaya might be caused by the lack of expression or decreased expression of some transcription factors and hormone-related genes, affecting hormone signal transduction or hormone biosynthesis. Analysis of aborted and normally developing pistil in papaya provided new insights into the molecular mechanism of pistil development and sex differentiation in dioecious papaya.

**Supplementary Information:**

The online version contains supplementary material available at 10.1186/s12864-021-08197-7.

## Background

Flowers are the reproductive organs of flowering plants, and their development involves complex regulatory processes. The precise control of the expression of flower development-related genes guides the formation of four whirls so that angiosperms exhibit a wide variety of flower morphologies [1]. The carpel is a unique reproductive structure in angiosperms that contains ovules and female gametophytes. The stigma at the top of the carpel facilitates pollen recognition and pollen tube elongation, and the carpel not only protects ovules from insects or microorganisms but also nurtures the development of plant male and female gametophytes. After fertilization, carpels develop into fruit.

Each step of flower organ development is genetically regulated, and a network of finely regulated genes directs the elaboration of flower morphology through temporal and spatial expression and interactions among specific genes [2]. Among these, the *AGAMOUS (AG)* gene plays a crucial role in the regulatory network of flower development [2]. AG is a C-class functional gene in the ABCDE model of flower development [3, 4] and plays an important role in regulating the development of stamens and carpel primordia [5]. *SEEDSTICK (STK)*, also known as *AGAMOUS-LIKE 11 (AGL11)*, is a D-class functional gene and transcription factor MADS-box family in the ABCDE model [6], which are essential for ovule development [7]. Silencing the homologs of *AG* and *STK* in poplars leads to changes in the characteristics of floral organs and affects the determination of floral organs, ovule differentiation, and seed hair development [8]. Further, *AGL11* also plays an essential regulatory role in ovule development and seed formation in tomato, grape, rice, and other species [9, 10]. *SUPERMAN (SUP)* is a regulator of flower homeotic genes and a transcription factor of the C2H2-type zinc finger motif [11]. The *SUP* gene in *Arabidopsis thaliana* determines the boundary between the third- and fourth-round organs and the growth of the integument, which is the cell layer surrounding the ovule [12]. *SlSUP* is a *SUP*-like gene that is expressed exclusively in female flowers and involved in ovule development in white campion *Silene latifolia* Poiret [13]. The function of *MdSUP11*, one of the 12 *SUPL* genes in apple (*Malus domestica* Borkh.), is related to vegetative and reproductive development in apples. Its overexpression affects the development of leaf and floral organs and plant height in tobacco [14].

The development of carpels and fruit are also directly or indirectly regulated by plant hormones. For example, in maize, a low concentration of jasmonic acid (JA) can promote the development of female organs and inhibit the development of male organs, resulting in abnormal development of seeds in tassels, which can be reversed by the application of JA [15, 16]. Ethylene can promote the development of cucumber carpels. In hermaphrodite cucumbers, a gene encoding ACC oxidase participates in carpel development by regulating the ethylene content, and ethylene-induced *ACS11* is also crucial for carpel development [17]. The numbers of ovules and seeds in Arabidopsis are positively regulated by brassinosteroid signaling [18]. Brassinosteroid controls the number of ovules by influencing the expression of the gene *BZR1* on ovule development. The *bzr1-1D* mutant (the gain-of-function of BZR1) increased the number of ovules and seeds, while the brassinosteroid-deficient mutant *det2-1* produced fewer ovules and seeds [19]. At the same time, brassinosteroids can regulate the number of ovules by down-regulating gibberellins biosynthesis, thereby promoting the stability of DELLA protein, which will eventually promote the initiation of ovule primordia in tomatoes [20].

Auxin is another critical plant hormone regulator of carpel development in flowers. In *Capsella bursa-pastoris* L., the polar distribution of auxin at the top of the carpel maintains a high concentration of auxin in the stigma, and this differential distribution of auxin promotes morphogenesis of the carpel [21]. Lateral transport of auxin can promote increased cell division and thereby cause the carpel to bend [22]. Also, decreased capacity for longitudinal polar auxin transport in the carpel can directly affect carpel length [23]. Further, auxin is distributed in a concentration gradient at different locations in developing plant structures. Thus, the observed differences in carpel morphology between *C. bursa-pastoris* and *Arabidopsis* are due to differences in the polar distribution of auxin during carpel development [21].

Dioecious plant species have been derived from hermaphroditic ancestral species in which a mechanism of stamen or pistil abortion evolved to prevent self-pollination. The resulting plant species with gynodioecious or androdioecious individuals eventually evolved into species with dioecious individuals [24]. During this evolutionary process, genes that regulate pistil or stamen abortion became the key genes for plant sex determination. Papaya (*Carica papaya* L.) is a rare trioecious plant species that can bear female, male, or hermaphrodite flowers on separate plants. Sex determination in papaya is controlled by pairs of sex chromosomes, for which XX represents female, XY represents male, and XY^h^ represents hermaphrodite plants [25] that originated about 7 million years ago. An 8.1 Mb sex-determination region has been detected in the Y^h^ chromosome (HSY), while the corresponding region in the X chromosome is 3.5 Mb in length [26]. Homologous sequence comparison between HSY and X reveals two evolutionary strata, corresponding to two chromosomal inversions on HSY that occurred 7 and 1.9 million years ago, respectively. The gene annotation of HSY revealed that there are 72 protein-coding genes, 50 of which have homologous alleles in the X corresponding homologous region, and the other 22 are HSY-specific genes. There are 84 genes in the X region with 34 X-specific genes [26, 27]. Similarly, a sex-determination region has also been detected on the Y chromosome (MSY) in male papaya, and the MSY and HSY regions share 99.6% similarity [28]. Papaya sex-determination genes are located within this region, as would be expected according to the theory of sex chromosome evolution in plants [29]. The female flowers of papaya have well-developed pistils and can accept pollen to form fruits. In contrast, male flowers cannot form fruits due to aborted pistil.

The pistils of papaya male and female flowers exhibit obviously distinct morphology, which provides an excellent opportunity to study the genetic mechanisms that control pistil development and sex determination in papaya. Here, RNA-Seq technology was used to analyze the pistil transcriptomes of female and male papaya flowers at different developmental stages. These data were used to determine the transcriptional differences between pistils in flowers of different sexes and to explore transcriptional regulation related to pistil development. We constructed a gene regulation subnetwork for carpel development in papaya based on the specificity of gene expression. Finally, candidate genes were identified for their possible important roles of auxin and brassinosteroid biosynthesis and signal transduction in the molecular mechanisms of sex differentiation and carpel development in papaya.

## Results

### Global transcriptome analysis of pistil development in female and male papaya flowers

**Fig. 1:**
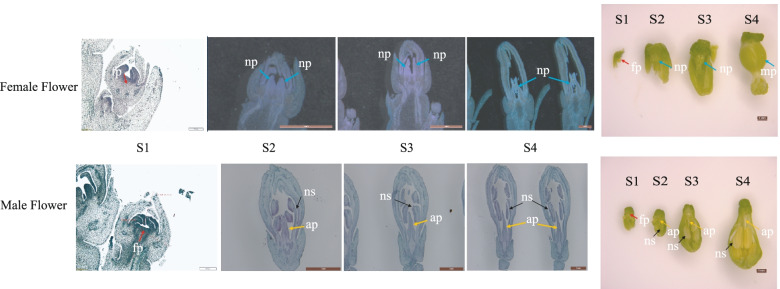
The phenotype of the papaya pistil at different developmental stages (S1 to S4) in female and male flowers. Abbreviations for the flower developmental stages: primordium formation stage (FS1 and MS1); pre-meiosis stage and post-meiotic stage (FS2, FS3, MS2, and MS3); mitosis stage (FS4 and MS4); F, Feamle; M, Male; fp, floral primordium; np, normal pistil; ap, aborted pistil; ns, normal stamen

Male papaya flowers bear an aborted pistil, while the pistils of the female flowers develop normally. Global transcription analysis of different developmental stages of female and male flower carpels can reveal the molecular mechanism of male flower carpel abortion. The development of papaya flowers was divided into four stages (Fig. 1). These four stages of pistil development represent major events, including flower primordium formation, carpel formation, meiosis, and mitosis. Stage 1 is the representative stage for flower primordium formation, the key stage for papaya sex determination when both male and female flowers are smaller than 1 mm in length. There is no difference in the morphology of male and female flowers in this stage (Fig. 1). Stage 2 and 3 flowers are 1-2.5 mm and 2.5-4.5 mm in length, respectively. At these stages, the male and female flowers form carpels with important morphological differences and continue to develop. Multiple normal carpels are formed in female flowers, while only one pointed carpel is formed in male flowers (Fig. 1). The Stages 2 and 3 of carpel development coincide with meiosis in papaya ovules. During stage 4, ovules and ovaries the 4.5-6 mm flowers enter the mitotic stage, and the carpels of the female flowers coalesce to form the normal pistil, while the male flowers abort pistil. A normal pistil is formed with an enlarged base during this stage in female flowers, of which stigma, style, and ovary are all developing normally, but a particularly slender aborted pistil is formed in male flowers (Fig. 1).

To detect candidate genes that might be involved in sex determination and carpel abortion in male papaya flowers, transcriptomes were analyzed from female and male flower primordia and carpels at these different developmental stages. The high-quality reads number in different pistil development stages ranged from 27 to 58 million in female flowers, and 34 to 90 million in male flowers. We also obtained 180/197 and 38/302 million high-quality reads on female and male leaves, respectively (Additional file 16). In all, more than 1.1 billion high-quality paired-end reads were obtained from different tissues, with an average of about 40 million reads per sample. The high-quality reads were mapped to the papaya reference genome. The range of mapped reads is 27 to 294 million, accounting for 93.13 to 98.01% of the total reads (Additional file 16). We used the Spearman correlation coefficient to estimate the consistency and repeatability of the sequencing samples. The closer the value is to 1, the stronger the correlation among replications. The Spearman correlation coefficient between expression values in different biological replicate samples varied from 0.94 to 0.98 in female flowers and from 0.92 to 0.99 in male flowers, indicating high-quality data replication (Additional file 1).

In total, about 91% of sequenced papaya genes were expressed in at least one of the ten samples. The percentage of genes expressed in different pistil development stages ranged from 80% (FS2) to 82% (FS1) in female flowers, and 82% (MS1) to 86% (MS3) in male flowers. From 4.18 to 5.37% of the genes showed extremely high (FPKM ≥100) transcript expression in the various tissues analyzed (Additional file 2a). The number of genes with high (50 ≤ FPKM < 100), medium (10 ≤ FPKM < 50), or low (1 ≤ FPKM < 10) transcript expression was similar in all pistil tissues (Additional file 2b). These analyses reflect effective transcriptome coverage of genes expressed during pistil development, which was beneficial for downstream analysis.

### Transcriptional analysis reveals relationships between different stages of pistil development in papaya

**Fig. 2:**
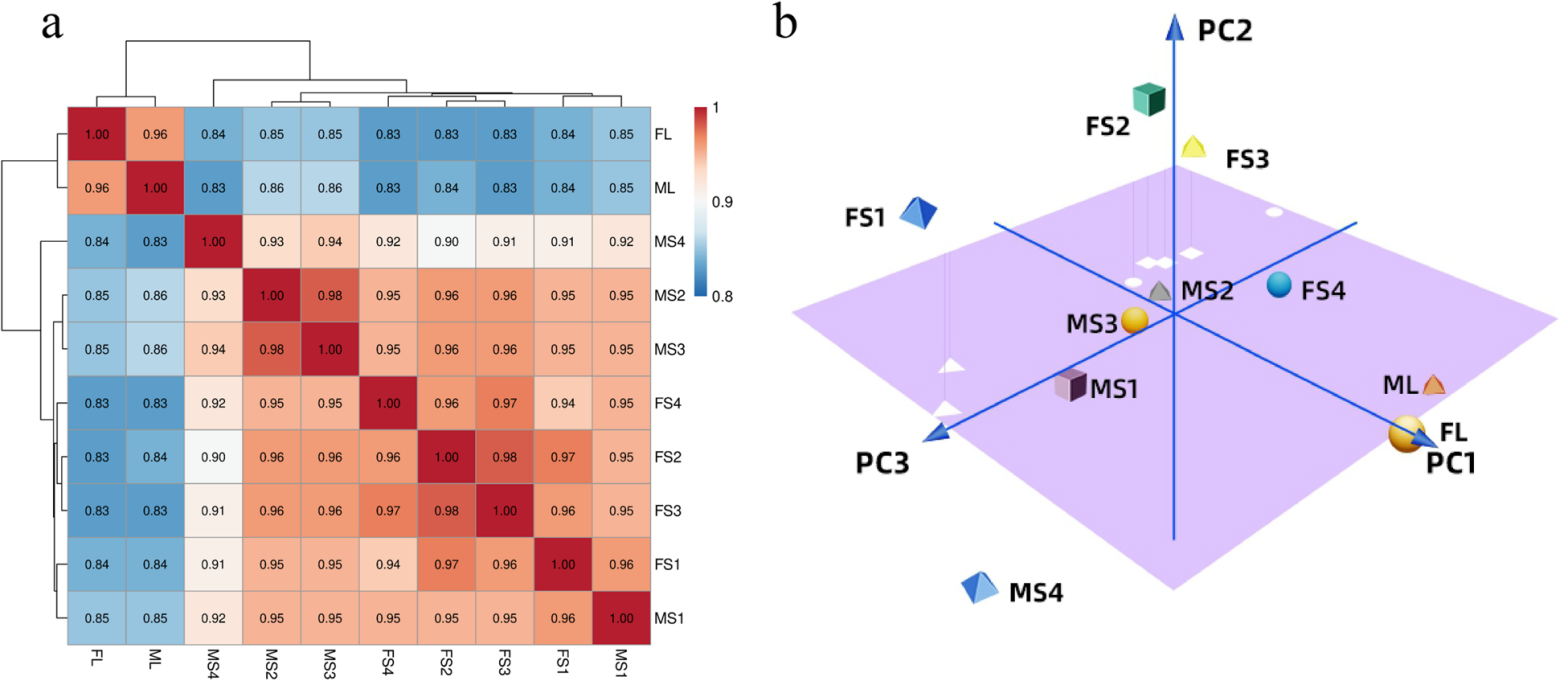
The transcriptional correlation of pistil at different developmental stages in female and male flowers. **a** SCC was performed to analyze transcripts expressed in leaves and at four developmental stages of the pistil in female and male papaya flowers, and the redder the color, the stronger the correlation. The legend on the right represents the correlation coefficient. **b** PCA cluster analysis of leaves and pistils in different stages, different development stages are represented by 3D shapes in different colors

To investigate global transcriptome differences during pistil development in female and male papaya flowers, PCA and SCC cluster analyses were performed based on the average FPKM value of all expressed genes in 10 samples. Higher correlations detected in these analyses might reflect more consistent transcriptional activity in various tissues or developmental stages. As expected, these analyses showed that the leaf transcriptomes of the male and female plants clustered together and differed significantly from the other tissues (Fig. 2). Significant differences in transcript abundances between different sexes at the early stage (FS1 and MS1) imply differences in transcriptional regulation activities in stage 1 between male and female flowers (Fig. 2b). The middle stages of pistil development (F/MS2 and F/MS3) showed very close clustering within sexes, indicating that their transcriptional programs were very similar at those times (Fig. 2b). Because meiosis is underway during both of these stages, combined them into one stage for downstream analysis. Interestingly, the transcriptional activity of the middle stage (F/MS2 and F/MS3) clustered more closely with that of the early stage (MS1) in male flowers, but more closely with that of the late-stage (FS4) in female flowers. In the later stage of pistil development (F/MS4), large differences in transcriptional activities were observed in male and female flowers (Fig. 2b). The large differences in transcription programs at F/MS4 might ultimately determine the morphology and fertility of pistils. Taken together, these results indicate that male and female papaya flowers exhibit greater differences in transcription programs at particular stages of pistil development, which helps to direct further exploration of the growth and development of pistils at the transcriptional level.

### Preferentially expressed genes during pistil development in papaya

**Fig. 3:**
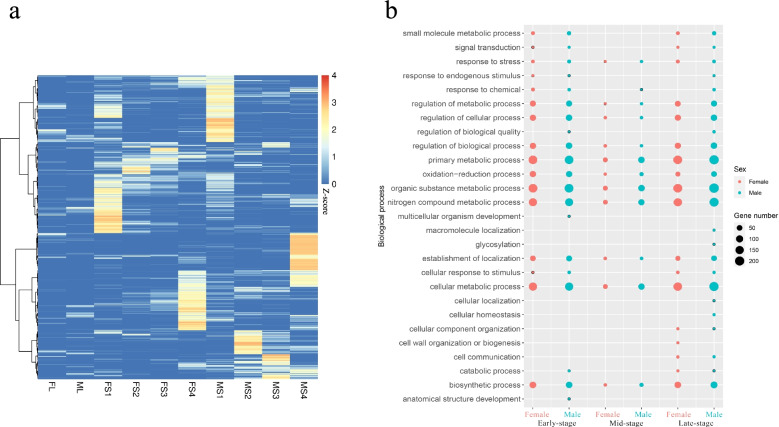
Preferential gene expression between male and female papaya flowers during different stages of pistil development. **a**, Heatmap of preferential gene expression at different stages of pistil development in female and male papaya flowers. The larger the Z-score value, the redder the color and the higher the gene expression **b**, The putative functions of genes preferentially expressed at different stages investigated by Gene Ontology (GO) term analysis. Different colors represent different sex types, and the size of the circle represents the number of genes

To investigate transcriptional activities during different stages of carpel development in male and female flowers, genes that are preferentially expressed at particular stages of carpel development were defined. A ‘stage-specific’ (SS) algorithm was used to define the genes preferentially expressed by each gender at a specific developmental stage. According to this criterion, the number of preferentially expressed genes were detected ranged from 125 to 911 in female flowers and from 223 to 982 in male flowers depending on the stage (Fig. 3a and Additional file 3). The differences in the profiles of preferentially expressed genes at each developmental stage suggest that different stages or samples exhibit independent growth and developmental processes.

Gene ontology (GO) term analysis of all the preferentially expressed genes revealed many common processes and few unique processes across stages of development (Fig. 3b). Unique processes include cellular organism development and the development of anatomical structures in male or female flowers during early development (F/MS1). The unique processes were found during late-stage development (FS4), including cell wall organization or biogenesis in the female flowers, macromolecule localization, glycosylation, cellular localization and cellular homeostasis in male flowers, and cellular component organization and cell communication in flowers of either sex. Genes expressed during the mid-stage of development were not enriched in unique GO terms.

Genes encoding 230 and 236 transcriptional regulators were uncovered in female and male flowers, respectively, of which 132 transcription factors (TFs) are shared by both transcriptomes (Additional file 4). The number of genes encoding TFs from one TF family (e.g., ERF, MADS, and HD-ZIP) expressed at different developmental stages varied between male or female flowers. Within a developmental stage, the number of expressed genes encoding TFs from one TF family differed between female and male flowers (Additional file 4). For example, the number of genes encoding MADS TFs expressed in female flowers was greater than that expressed in male flowers in the early stage (MS1), while genes encoding MYB TFs showed the opposite expression pattern. The fewer TFs were preferentially expressed in the middle stages (F/MS2 and F/MS3) of carpel development were detected, implying that the transcriptional program for TF-encoding genes is not very active at that time.

To verify the accuracy of our stage-specific (SS) expression algorithm, we randomly chose 24 preferentially expressed genes and performed quantitative reverse transcription PCR (RT-qPCR) experiments to analyze their expression at different developmental stages in females and males flowers. RT-qPCR verification of the expression of these genes showed that 19 (79.2%) of the SS genes could be verified as consistent with the results of our RNA-Seq analysis (Additional file 5).

### Analysis of differentially expressed genes and construction of a gene regulatory network for papaya flower pistil development

To study the transcript differences characteristic of different stages of female and male flower development in papaya, differentially expressed genes (DEGs) in each stage were identified using DESeq [30] and edgeR [31] (Additional file 6). In total, we identified 7165 and 8074 DEGs using DESeq and edgeR, respectively. A total of 7058 DEGs were shared between both male and female tissues, including 1397 in FS1_VS_MS1; 1403 in FS2_VS_MS2; 1386 in FS3_VS_MS3; and 2872 in FS4_VS_MS4. These shared DEGs were then subjected to gene regulatory network (GRN) analysis. The analysis of the gene regulatory networks during pistil development in different sex type flowers provided a clearer understanding of the occurrence and regulation of the pistil development process. Based on the results of PCA analysis and RNA-Seq of tissue sections, S2 and S3 as the meiosis stage were combined together for further analysis.

The distribution of differentially expressed TF (DETF) families at particular stages was characterized (Additional file 7). During the FS1, FS2-3, and FS4 stages, 73, 104, and 140 up-regulated DETFs were identified, respectively, while during the MS1, MS2-3, and MS4 stages, 29, 112, and 116 up-regulated DETFs were found, respectively. Notably, the top three gene families encoding DETFs were the bHLH, MYB, and MADS-box families. Interestingly, some of these TF-encoding gene families were expressed only in male or female flowers. Transcripts of genes encoding members of the ARR-B, B3, C3H, E2F/DP, ERF, GATA, GRF, HB-other, LFY, LSD, NF-YA, SAP, and Whirly TF families were expressed in female flowers but were not in male flowers, while genes encoding members of the CAMTA, NF-YC, and Nin-like TF families expressed in male flowers but not in female flowers.

To study the GRNs that operate during papaya pistil development, co-expression networks for both female and male flowers at three different developmental stages were constructed using Pearson correlation coefficient (PCC) and mutual rank (MR) analyses. Only DEGs with PCC and MR values above 0.95 were included in the network construction to avoid making spurious associations. A motif enrichment analysis on the 2 kb upstream region of each gene was performed to predict possible regulatory interactions between the gene and TFs that bind to enriched cis-regulatory elements in its promoter region [32]. Network membership varied between male and female papaya flowers at different stages of development and generally increased over-development (Additional files 8, 9 and 10). Network size also varied between sexes at different stages, in which the male papaya flower network contained more co-expressed and co-regulated genes than the female flower network during the primordial (FS1) and mitotic (FS4) stages (Additional files 8 and 10). However, there were more co-expressed and co-regulated genes in the female network during the meiosis (FS2-3) stage (Additional file 9). The average clustering coefficient, average path length, average degree, average connectivity, and total nodes of the papaya network for each period are shown in Additional file 11. The number of nodes and edges of the mitotic stage (F/MS4) network was much greater than that of the primordium stage (F/MS1) and the meiosis stage (F/MS2-3) network. In addition, the FS4 stage has the highest average degree and connectivity (Additional file 12), suggesting the existence of a more complex GRN than others.

GO term enrichment analysis of the genes in each network revealed key biological processes taking place during each stage of pistil development (Additional file 13). In female flowers, the genes associated with the primordium (FS1) stage were enriched for GO terms such as ‘response to high light intensity’, ‘response to heat’, and ‘response to abiotic stimulus’. GO term enrichment analysis of genes expressed in female flowers during the meiosis (FS2-3) and mitotic (FS4) stages identified enrichment of many flower development-related GO terms such as ‘carpel morphogenesis’, ‘floral organ morphogenesis’, ‘meristem development/maintenance’, ‘organ morphogenesis’, and ‘plant organ development’. In male flowers, GO terms such as ‘extracellular region’, ‘cell wall’, and ‘tryptophan metabolic process’ became enriched during the primordium (MS1) stage. Genes associated with meiosis (MS2-3) and mitotic (MS4) stages showed enrichment of GO terms such as ‘response to biotic stimulus’, ‘external encapsulating structure’, ‘cation transmembrane transport’, and ‘ion transport’.

### Construction of a coregulatory subnetwork for pistil abortion

**Fig. 4:**
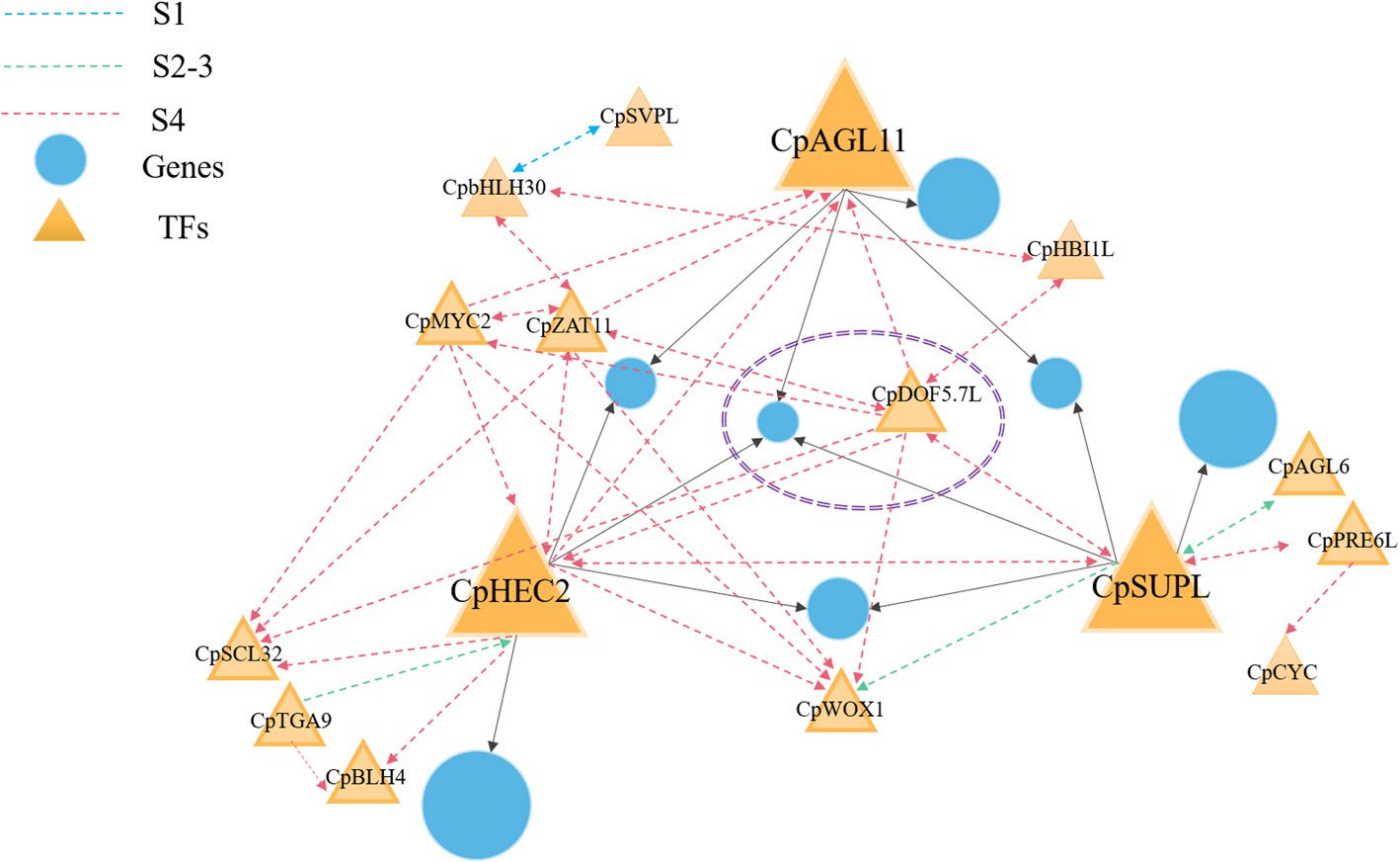
The *CpAGL11-CpSUPL-CpHEC2* regulatory subnetwork. The size of each blue circle represents the number of genes. The size of each triangle represents the importance of a transcription factor in the network, i.e., the degree of the node. The regulation between transcription factors is represented by dotted lines, and the solid black lines show the regulation of transcription factors on other genes. The arrow points represent the regulatory relationship between genes, and different colors are used at different developmental stages

To further explore the functions of important genes in the network, nodes with degree values in the top 10% were selected as candidate hub genes for further analysis. When the degree value of a gene is higher, it indicates that it is higher connectivity in the network, potentially indicating its importance in the regulatory network structure. A total of 1297 candidate hub genes were identified in six networks, including 70 in FS1, 122 in FS2-3, 587 in FS4, 11 in MS1, 248 in MS2-3, and 259 in MS4, of which 1056 genes are unique and 117 genes share at least two networks. Then, genes specifically expressed in female or male flowers at three different stages were identified (0 < FPKM < 1 = no expression, FPKM > 1 = expression). 18 TFs as female-specific expressed genes and 7 TFs as male-specific expressed genes were identified that could play important roles in sex-specific regulation of pistil development (Table 1 and Additional file 15). We also investigated the expression of these 25 TFs in 1-6 mm mixed flowers of different sex types [33]. Among them, the expression patterns of 18 TFs are consistent, and the remaining 7 TFs are expressed in mixed flowers of females and males. This indicates that these TFs specifically expressed in the primordium stage may also are involved in the growth and development of other floral organs except besides carpels (Additional file 15). A subnetwork was constructed using the network neighborhood around these key TF nodes. The *CpHEC2*, *CpSUPL*, and *CpAGL11* genes were expressed specifically during all three stages in females and were assigned a central role in the subnetwork (Fig. 4). Experimental verification of the expression of these three genes in female flowers by RT-qPCR was consistent with the results of our RNA-Seq data analysis (Additional file 14). The combined functions of individual TFs, any interconnected TFs, and protein-coding genes should be taken into account when describing the regulation of flower development.

**Table 1:** Information of stage-specific expression hub genes in the female and male gene regulatory network

Gene ID	Female	Male	Symbol	Arabidosis homolog	Stage	Function
sunup.05G0006810	EXP	NO	CpAGL6	AT2G45650	FS2-3	Flowing time
sunup.04G0000360	EXP	NO	CpHEC2	AT3G50330	FS1/FS2-3/FS4	Gynoecium development
sunup.03G0006060	EXP	NO	CpSUPL	AT3G23130	FS1/FS2-3/FS4	Carpel development
sunup.01G0026370	EXP	NO	CpAGL11	AT4G09960	FS1/FS2-3/FS4	Carpel and ovule development
sunup.01G0022430	EXP	NO	CpSVPL	AT4G24540	FS1	Flowing
sunup.02G0012880	EXP	NO	CpTGA9	AT1G08320	FS2-3/FS4	Flower development
sunup.01G0000330	EXP	NO	CpCYC	AT1G67260	FS2-3/FS4	Longitudinal elongation of petioles, rosette leaves and inflorescent stems
sunup.04G0001590	EXP	NO	CpDOF5.7 L	AT5G65590	FS4	Guard cell differentiation
sunup.04G0003750	EXP	NO	CpHBI1L	AT2G18300	FS4	Cell elongation and proliferation
sunup.03G0023770	EXP	NO	CpZAT11	AT2G42410	FS4	Flower growth and development
sunup.05G0010320	EXP	NO	CpMYC2	AT4G00870	FS4	Jasmonate-Mediated Growth
sunup.08G0004940	EXP	NO	CpATH51	AT5G03790	FS2-3/FS4	Organ proportions
sunup.02G0001210	EXP	NO	CpbHLH30	AT1G68810	FS4	Leaf development
sunup.02G0008550	EXP	NO	CpWIP6	AT1G13290	FS4	Shoot and root development
sunup.02G0002680	EXP	NO	CpPRE5L	AT3G28857	FS2-3/FS4	Cell Elongation and Plant Development
sunup.09G0005320	EXP	NO	CpWOX1	AT3G18010	FS2-3/FS4	Leaf blade outgrowth and floral organ development
sunup.03G0015290	EXP	NO	CpSCL32	AT3G49950	FS4	Regulate growth and flowering
sunup.04G0013610	EXP	NO	CpBLH4	AT4G36870	FS2-3/FS4	Ovule morphogenesis
sunup.03G0020730	NO	EXP	CpMUTE	AT3G06120	MS2-3	Stomatal development
sunup.06G0008830	NO	EXP	CpAMS	AT2G16910	MS2-3	Tapetal cell development
sunup.01G0006170	NO	EXP	CpBZIP43L	AT5G38800	MS1/MS4	NA
sunup.08G0003710	NO	EXP	CpNAC90L	AT3G44350	MS4	NA
sunup.02G0022760	NO	EXP	CpDIV	AT5G58900	MS1/MS4	Vascular Development
sunup.06G0024960	NO	EXP	CpMYB5	AT3G13540	MS1/MS4	Seed coat and endosperm layers formation

**Fig. 5:**
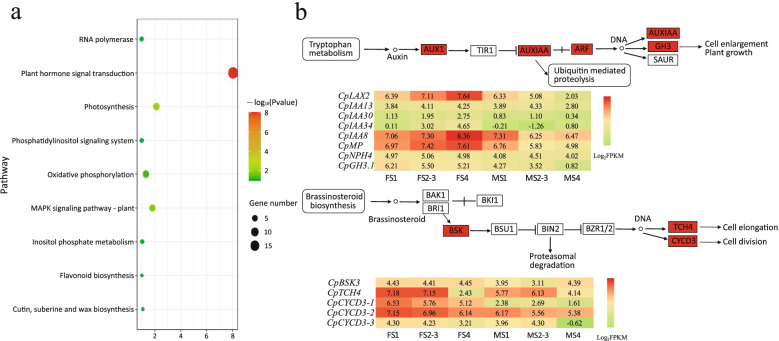
Functional enrichment analysis of the *CpAGL11-CpSUPL-CpHEC2* regulatory subnetwork. **a**, KEGG pathway enrichment analysis of the *CpAGL11-CpSUPL-CpHEC2* regulatory subnetwork. **b**, Gene term Analysis of auxin/brassinosteroid signal transduction pathway [34]. The upper half is the transduction pathway of plant hormones; the red rectangle indicates the enriched difference items; and the lower half is the corresponding differential gene expression heatmap

**Fig. 6:**
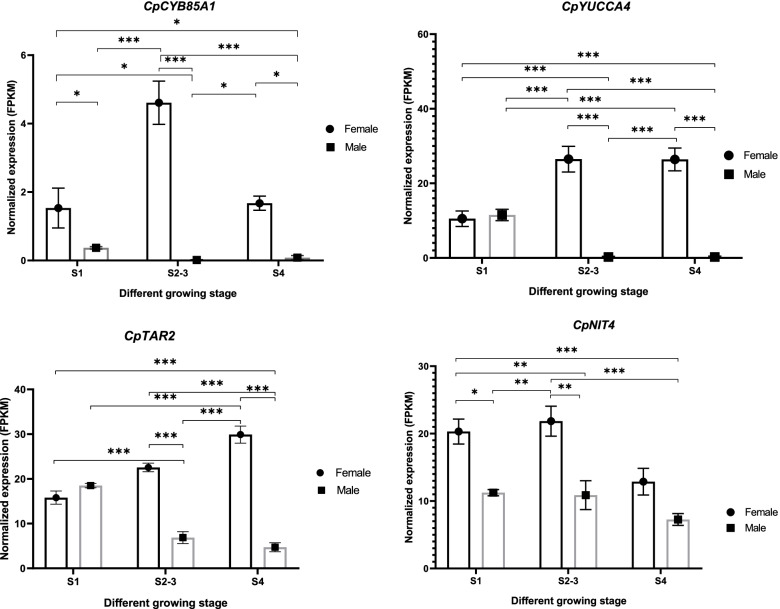
Comparison of transcript expression of auxin and brassinosteroid synthesis-related genes at different developmental stages in male and female papaya flowers. Mean RPKM (±SD) in female and male papaya flowers at different development stages (Tukey’s HSD, *p*-value = * < 0.05, ** < 0.01, *** < 0.001)

KEGG pathway analysis of the *CpHEC2*, *CpSUPL*, and *CpAGL11* subnetwork genes revealed that expressed genes were enriched for pathways related to plant hormone signal transduction (Fig. 5a). Exploration of plant signal transduction pathways revealed differentially expressed genes associated with tryptophan metabolism (auxin) pathways and brassinosteroid signal transduction pathways (Fig. 5b). In the tryptophan metabolism pathway, *CpLAX2, CpIAA8*, and *CpMP* showed higher expression in females than in males and their expression gradually increased during each of the three successive stages of pistil development. In the brassinosteroid biosynthesis pathway, the overall expression of *CpBSK3*, *CpTCH4* and *CpCYCD3* was also higher in females than in males (Fig. 5b and Additional file 15). Further, we analyzed differences in the expression of genes related to auxin and brassinosteroid biosynthesis using Tukey’s HSD and found significant differences in the expression of *CpCYB85A1, CpYUCCA4, CpTAR2* and *CpNIT4* between male and female flowers at different stages of development (Fig. 6 and Additional file 15). *CpCYB85A1* catalyzes the last reaction to produce brassinolide and converts 6-deoxycasterone into a seven-membered lactone ring [35]. *CpNIT4* encodes a gene essential for the transformation of indole-3-acetonitrile (IAN) into indole-3-acetamide (IAM) and indole-3-acetic acid (IAA) [36]. *CpYUCC4* encodes a flavin monooxygenase, which is a possible bridge between auxin biosynthesis and pistil development [37, 38]. In summary, we suggest that papaya pistil abortion might be caused by the lack of expression of some important TFs such as *CpHEC2*, *CpSUPL* and *CpAGL11* that are mainly involved in the auxin and brassinosteroid synthesis pathways.

## Discussion

The construction of GRNs is useful for depicting gene interactions, and can also be used to predict possible regulatory relationships and modules within the network [39]. By performing transcriptomic analysis of male and female flowers from three stages in papaya, we constructed a series of networks underlying flower development in papaya. GO enrichment analysis of network members revealed that genes in the primordium stage were enriched mainly in some response-related pathways, such as light, heat and abiotic stimulus (Additional file 13). However, we did not identify any other genes or pathways in this stage that seemed to be related to sex determination. The results suggested that sex determination in female papaya flowers might already be established before the primordium stage as there was no trace of stamens, whereas sex determination in male flowers occurred at a later stage when the pistil development was aborted. At the S2-3 and S4 stages in females, many significantly enriched GO terms such as ‘carpel morphogenesis’, ‘floral organ morphogenesis/development’, ‘plant organ development’, and ‘organ morphogenesis’ were related to floral organ formation and development (Additional file 13). Importantly, genes involved in these pathways exhibited decreased expression or no expression in males (Table 1), suggesting that processes leading to pistil abortion in male flowers might begin at the meiosis stage or earlier.

TFs are essential regulators for the transcriptional control of gene expression, which is crucial for the development and growth of plants. Previous studies in various species discovered many TFs that are involved in plant growth, organ development, and response to biotic and abiotic stresses [40–42]. The identified several candidate TFs that might be crucial for the development of papaya flowers (Fig. 4 and Table 1). For instance, *CpHEC2* is a homolog of the *Arabidopsis thaliana* gene *HEC2* (*AT3G50330*), which belongs to the basic helix-loop-helix (bHLH) TF family. In *Arabidopsis*, *HEC* and *SPT* act synergistically to regulate the development of the female reproductive tract and are probably involved in auxin-mediated control of gynoecium patterning. Loss of *HEC* gene function can result in defective transmitting tract, stigma, and septum, as well as reduced fertility [43]. Further, *CpSUPL* and *CpAGL11* have already been shown to play an important role in carpel development in flowers [8, 11, 13]. Additionally, the other differentially expressed TFs in female papaya flowers were mainly related to the regulation of flowering. Taken together, these female-specific TFs might play a pivotal role in regulating the differential floral development patterns of male and female papaya flowers.

Plant hormones act as signaling compounds that promote and regulate plant growth and development. Phytohormones influence many processes of floral and post-floral morphogenesis in angiosperms [44]. Various plant hormone signaling pathways control flower development by transmitting both endogenous and environmental signals to genes that directly control flowering [45]. The significant differences between males and females in the expression of genes related to plant hormone signal transduction suggested that the inhibition of plant hormone signal transduction might be an important cause of pistil abortion in male flowers (Fig. 5). In addition, some genes related to auxin and brassinosteroid synthesis are not expressed or are expressed at lower levels in male flowers were detected, in which pistils abort (Fig. 6). These findings provide a basis for further functional studies of plant hormone function in pistil development, which will improve our understanding of the molecular mechanisms driving the regulation of pistil development in papaya.

## Conclusions

Discovering mechanisms for pistil formation and development has implications in dioecious crop improvement. The normal development of the pistil is essential to the reproduction of flowering plants. Papaya is a dioecious plant, but there is an aborted pistil in male flowers that can be restored sporadically under optimal environmental conditions. In this study, we divided the pistil into four developmental stages. The first stage (F/MS1) is the primordia period, the second and third stages (F/MS2 and F/MS3) are the meiotic period, and the fourth is the mitotic period. The transcriptome analysis of these stages revealed differentially expressed regulatory genes in transcription levels. A transcription factor regulatory network *CpAGL11-CpSUPL-CpHEC2* was constructed. The specific expression of some transcription factors closely related to pistil development in female flowers also were detected. Further functional analysis showed that the differential gene expression in phytohormones signaling pathway (mainly auxin and brassinosteroid) and auxin/brassinosteroid biosynthesis could be the main reason leading to pistil abortion.

## Methods

### Morphological observation and sampling of the pistil development stage

Female and male flowers of papaya were sampled and fixed according to flower size and length and stored in FAA liquid. After putting samples through an alcohol dehydration series, flower samples were embedded in paraffin LEICA EG 1150 H) in a Leica EG 1150 H paraffin embedding apparatus (Leica, Germany) at a melting point of 50-52 °C. Transverse sections with a thickness of 10 μm were made on a Leica automatic rotary microtome and stained with Brilliant Green (BIO-RAD). Sections were observed and imaged under an Olympus Model BH2 optical microscope (Olympus, Japan). For the specific methods used for paraffin embedding and sectioning, please refer to [46].

The sectioned and embedded papaya flowers were dissected under a dissecting microscope (Olympus, Japan) to isolate the different developmental stages of the male and female papaya flowers. Due to the difficulty in sampling pistils from male and female flowers at the primordium stage, the flower buds at the primordium stage was used as the experimental material for this study. Each sample comprised three biological replicates, each consisting of multiple pistils.

### RNA extraction, library construction and sequencing

The different tissue samples were placed in TRIzol® Reagent (Invitrogen), and total RNAs were extracted according to the TRIzol manufacturer’s instructions. Residual genomic DNA (gDNA) in the RNA samples was then removed by DNase treatment (DNase I RNase-free, Thermo Scientific, USA). An Agilent 2100 BioAnalyzer (Agilent Technologies) was used to determine the quality and integrity of RNA. High-quality RNA samples with no signs of degradation are used to prepare RNA-Seq libraries.

Each RNA-Seq library was constructed separately using the NEBNext Ultra RNA Library Prep Kit for Illumina (New England Biolabs) according to the manufacturer’s protocol. The library was evaluated by electrophoresis (0.8% agarose, TBE 1X buffer), and quantified with a fluorometer (Qubit reagent fluorometer, Invitrogen, USA). A total of 18 cDNA libraries were sequenced on an Illumina platform (HiSeq2500) to generate a larger number of paired-end sequence reads (150 nt). Raw RNA sequence data for each library is publicly available on NCBI BioProject, under the accession number: PRJNA687615 (https://www.ncbi.nlm.nih.gov/bioproject/PRJNA687615). Besides these libraries, RNA sequencing data from normal female buds (Accession number: PRJNA532376), normal female leaves (Accession number: SRX6473537 and SRX6473538), normal male buds (Accession number: PRJNA532376), and normal male leaves (Accession number: SRX6473540 and SRX6473541), were downloaded from the Sequence Read Archive (SRA) database [47]. The information of the analyzed library is presented in Additional file 16.

### Read mapping and gene expression analysis

The raw data were filtered by removing adapters and low-quality bases using Trimmomatic [48]. The clean high-quality reads were then mapped to the papaya reference genome [49] using the Bowtie2 [50] with the end-to-end model default parameter setting. FPKM (Fragments Per Kilobase pair per Million reads) values were calculated using RSEM [51]. The SCC algorithm was used to calculate the correlations between experimental values obtained from two or three biological replicates of samples. PCA analysis was performed using the R function prcomp and plotted using the pca3d package [52], respectively.

In this study, Z-score was used to represent relative gene expression [53]. The Z-score of the gene *i* in the stage or sample *j* is determined by the equation *z*_*ij*_ *= (x*_*ij*_ *-* μ_i_*)/SD*_*i*_, where x is the expression value FPKM, and *μ* and *SD* are the mean and standard deviation of the sample, respectively.

### Identification of preferentially expressed genes

The preferentially expressed genes in different stages or samples were defined using the SS scoring algorithm [54, 55], which compares the expression of a gene in a specific stage or sample with its maximum expression level in all other stages or samples. The expression value of a gene *i* in 10 samples is denoted as *EV*_*i*_ = (*E*^*i*^_*1*,_ *E*^*i*^_*2*,_ *E*^*i*^_*3*,_ *E*^*i*^_*4*…,_ *E*^*i*^_*9*,_ *E*^*i*^_*10*_), so the SS score of gene *i* in sample or stage *j* was calculated as: *SS(i,j)* = 1 - (max *E*^*i*^_*s*_/*E*^*i*^_*j*_), where 1 ≤ *s* ≤ 10, *s* ≠ *j*. The higher the SS score of a gene at a particular stage, the higher the possibility of preferential expression of the gene at this stage. In this study, a gene with an SS score greater than 0.3 was defined as preferentially expressed in that sample or stage.

### Analysis of differential gene expression

The DESeq2 [30] and edgeR [31] with default parameters in R were used to calculate differential expression of genes. The differential expressed genes (DEGs) were designated as those with FDR adjusted *p* < 0.05 and |log2 fold change| > 1.

### GO term and KEGG pathway enrichment analysis

Subsequently, analysis of the potential functions of differentially expressed genes was performed in two ways. Gene ontology enrichment analysis was performed using TBtools [56]. The *p*-value was calculated for each enriched GO term, and terms with *p*-value ≤0.05 were considered meaningfully enriched. KEGG pathway enrichment analysis was performed using the R2HTML package in R. Similarly, *p*-value ≤0.05 was also the criterion for meaningful pathway enrichment. The bubble charts for GO term and KEGG pathway enrichment were drawn using ggplot2 in the R package.

### Gene co-expression construction

Mutual rank (MR) analysis [57] was used to identify significant co-expression relationships between DEGs in papaya pistils. Pearson Correlation Coefficients (PCC) were determined for all DEGs from papaya pistils in this study using R. The stringent criterion for designating positively or negatively correlated gene expression was set at ≥|0.95|. The MR was calculated from the PCC rank, and geometric means were taken between the PCC rank of gene A to gene B and that of gene B to gene A. MR value ranged from 0 to 1, with 1 representing the most significant interaction. All MR values of > = 0.95 for interactions were included in construction of a co-expression network.

PlantPAN [58] was used to identify genes encoding transcription factors in the whole papaya genome and detected a total of 1475 genes encoding putative transcription factors for downstream analysis. In this study, the promoter sequences were defined as the 2 Kb upstream of the predicted Transcription Start Site (TSS) for every gene in the papaya genome were retrieved from papaya reference genome [49]. The database of known transcription factor binding sites at JASPAR Plantae [59] (http://jaspar.genereg.net/) and the PlantTFDB motif database [60] (http://planttfdb.gao-lab.org/) were used to predict the *cis*-regulatory elements (CREs) in target gene promoter regions. Promoter sequences of DEGs were analyzed for statistical overrepresentation of CREs for particular TF families using a MEME suite tool called AME [61]. The significance of motif enrichment was tested using Fisher’s exact test with a Bonferroni correction for multiple comparisons. A *p*-value < 0.001 for a candidate motif was considered a meaningful enrichment, so that CRE would then be considered included in construction of the gene regulatory network. The final networks contained highly significant interactions (MR > = 0.95) between pairs of differentially expressed genes with predicted binding interactions between TF and target genes (DEGs). The resulting network was visualized using Cytoscape version 3.6.1 [62].

### RT-qPCR analysis

We used RT-qPCR to validata false positives of RNA-Seq analysis. The primers were designed based on gene model sequences (Additional file 17). The 1 μg RNA as a template for reverse transcription using PrimeScript First Strand cDNA Synthesis Kit (TaKaRa). Then the synthesized first-strand cDNA was diluted 10-fold, and 1 μl of the diluted cDNA was used for real-time quantitative PCR (qPCR reaction). The papaya TATA binding protein 2 (TBP2) genes were included as internal reference genes for normalization [63]. Each RT-qPCR analysis was performed in triplicate. The 2^−△△Ct^ method was used to calculate the relative expression [64].

### Method declaration

All analysis methods are implemented in accordance with relevant guidelines and regulations in this manuscript.

## Supplementary Information

**Additional file 1.** The heatmap of the biological repeat correlation analysis of RNA-seq data samples. The suffixes 1, 2, and 3 represent three repeats, and the leaf sample has only two repeats. F, Female; M, Male; S, stage; L, leaf; FS1, Female stage1; and so on.

**Additional file 2.** Global transcript analysis in different stages/samples. a, Count the expression ratio of high/medium/low expressed genes. Percentage is used to indicate the number of genes at different expression levels b, Comparison of the number of expressed genes between females and males. The height of the histogram represents the number of gene expressions in different stages.

**Additional file 3.** Statistics of genes preferentially expressed by different sexes at different developmental stages.

**Additional file 4.** Statistics of transcription factors preferentially expressed in different stages.

**Additional file 5.** Comparison of gene expression in RT-qPCR experiment and RNA-seq data analysis. a, Female samples. b, Male samples.

**Additional file 6.** Count the differentially expressed genes of different methods.

**Additional file 7.** Bar chart of differentially expressed transcription factors between females and males at different stages. The abscissa shows the different transcription factor families. Different colors represent different stages of pistil development.

**Additional file 8.** Diagram of gene regulation network in primordium stage (S1), red triangles represent transcription factors, blue circles represent other genes. The black lines link the regulatory relationships between genes a, Female sample. b, Male sample.

**Additional file 9.** Diagram of gene regulation network in meiosis stage (S2-3), red triangles represent transcription factors, blue circles represent other genes. The black line represents the regulatory relationship. a, Female sample. b, Male sample.

**Additional file 10.** Diagram of gene regulation network in mitosis stage (S4), red triangles represent transcription factors, blue circles represent genes. Transcription factors and regulated genes are connected with black lines. a, Female sample. b, Male sample.

**Additional file 11.** Statistics of topological attribute information of different gene regulatory networks.

**Additional file 12.** Statistics information of each node in the network from six gene regulation networks.

**Additional file 13.** Bubble chart of GO enrichment analysis of gene regulatory network in different stages. Different bubbles visually show the sharing and unique pathways of different networks.

**Additional file 14.** RT-qPCR to verify the expression pattern of CpHEC2, CpSUPL, and CpAGL11 genes. The Y coordinate represents the normalized expression, and the X coordinate represents different developmental stages.

**Additional file 15.** The expression information of important genes at different developmental stages in this study.

**Additional file 16.** Summary statistics of RNA-Seq data from papaya male and female flowers.

**Additional file 17.** Primer information used for RT-qPCR experimental verification in this study. Primers CpHEC2, CpSUPL, and CpAGL11 are used to verify the false positive of RNA-seq differential expression analysis. FS and MS primers are used to verify the false positives of the female and male preferential expression algorithm, respectively.

## Data Availability

The pistil RNA-seq raw data was uploaded to NCBI BioProject, under the accession number: PRJNA687615 (https://www.ncbi.nlm.nih.gov/bioproject/PRJNA687615).
