# Supplementary figures and images for: Gene regulation network analyses of pistil development in papaya
Source: BMC Genomics. 2022 Jan 5;23:8. doi: 10.1186/s12864-021-08197-7 (PMC8729085; doi:10.1186/s12864-021-08197-7)

a

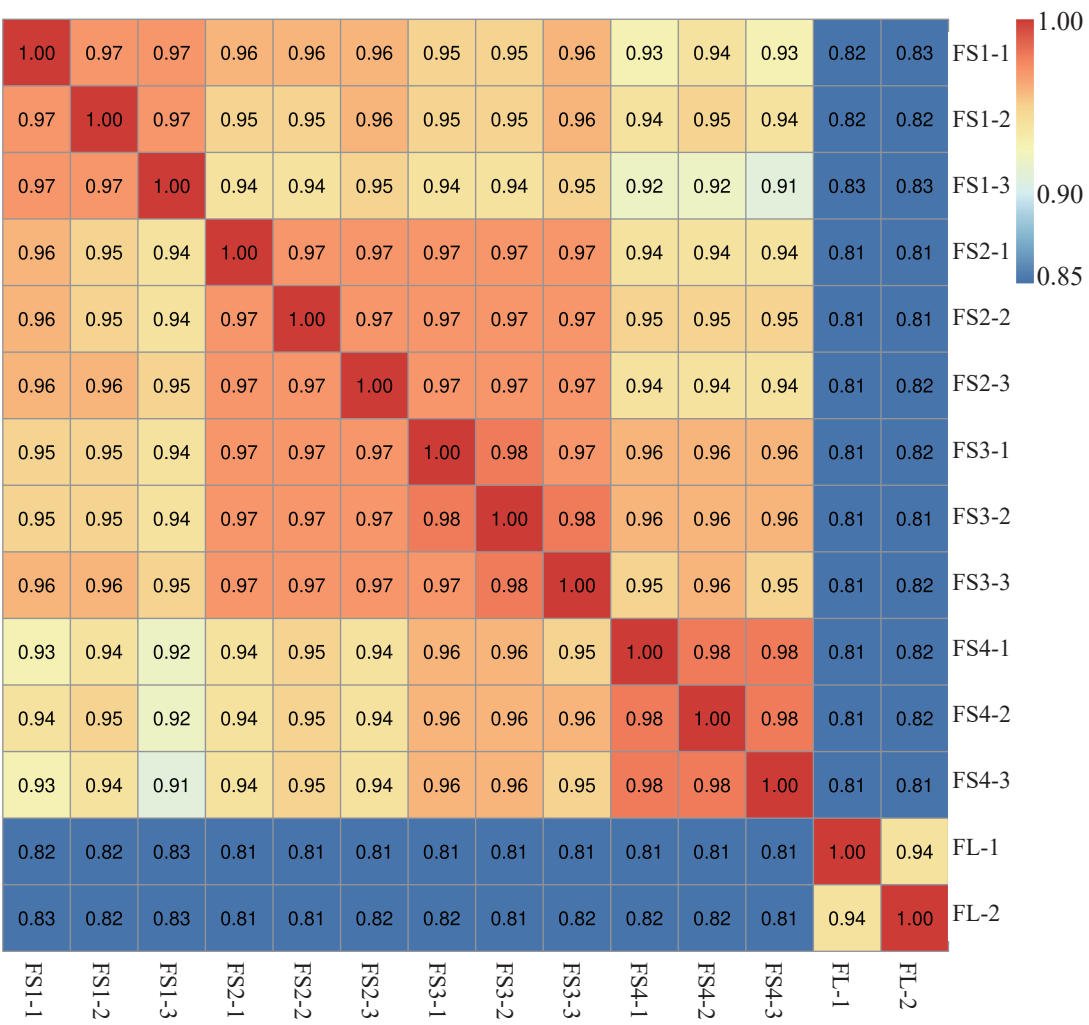

b

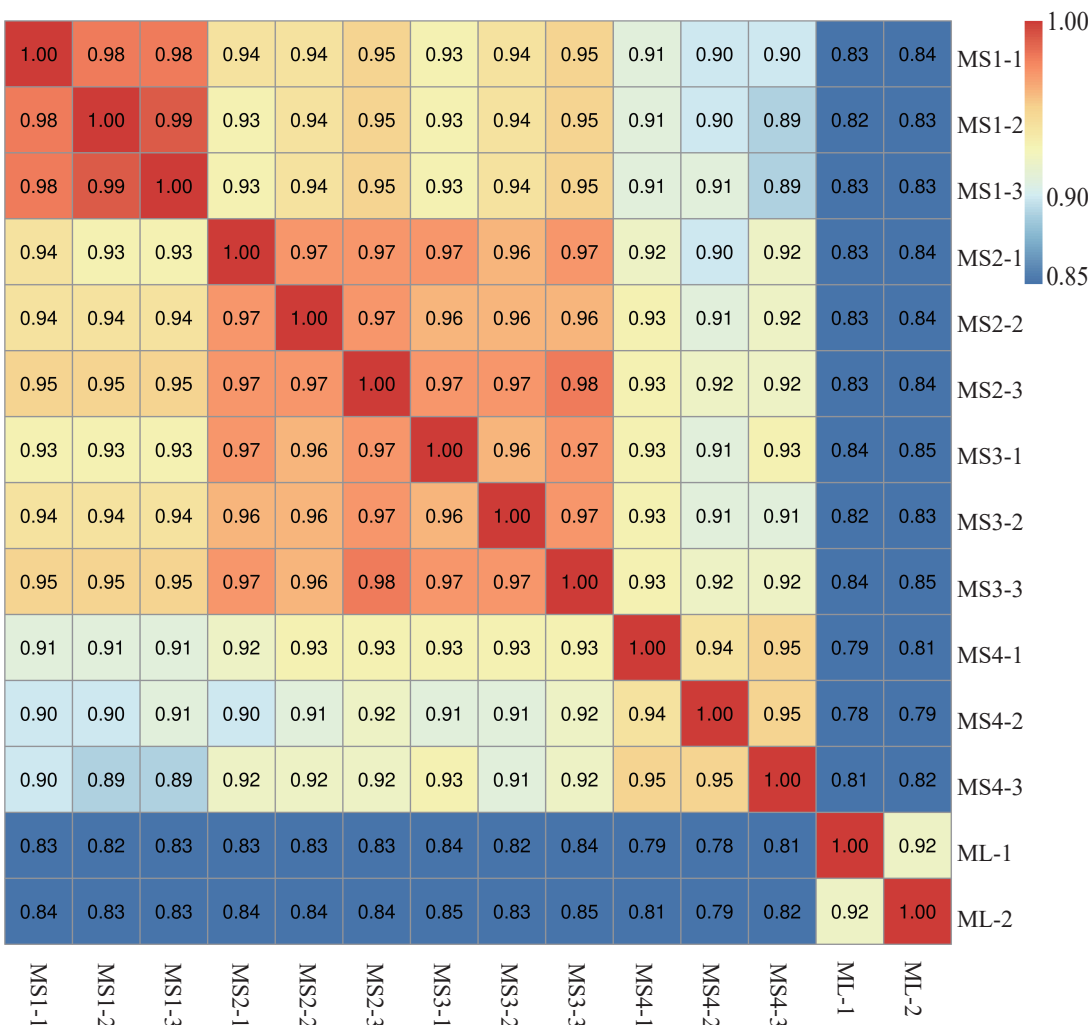

Supplement: Supplementary file 1 — Additional file 1. The heatmap of the biological repeat correlation analysis of RNA-seq data samples. The suffixes 1, 2, and 3 represent three repeats, and the leaf sample has only two repeats. F, Female; M, Male; S, stage; L, leaf; FS1, Female stage1; and so on. [file 12864_2021_8197_MOESM1_ESM.pdf]

a

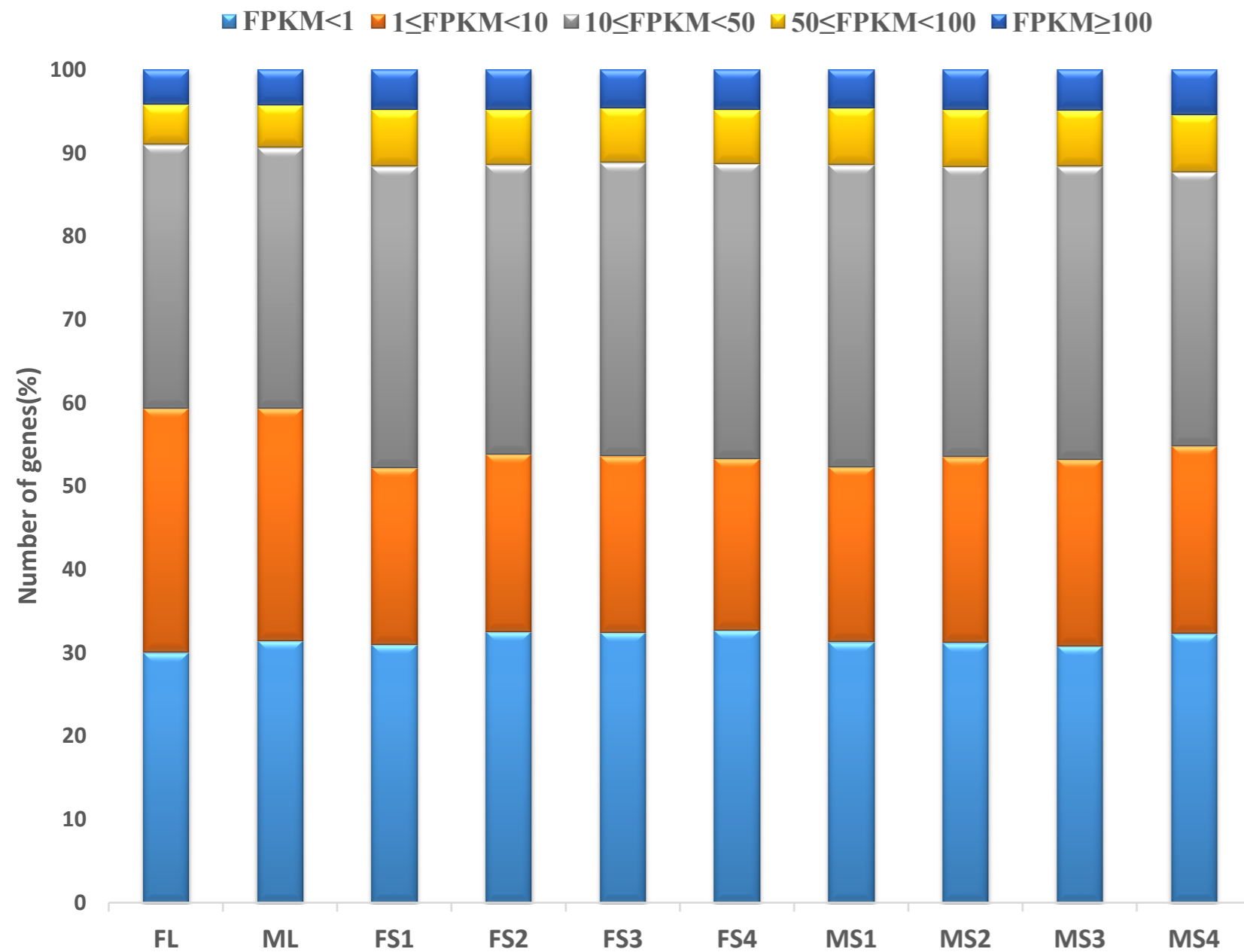

b

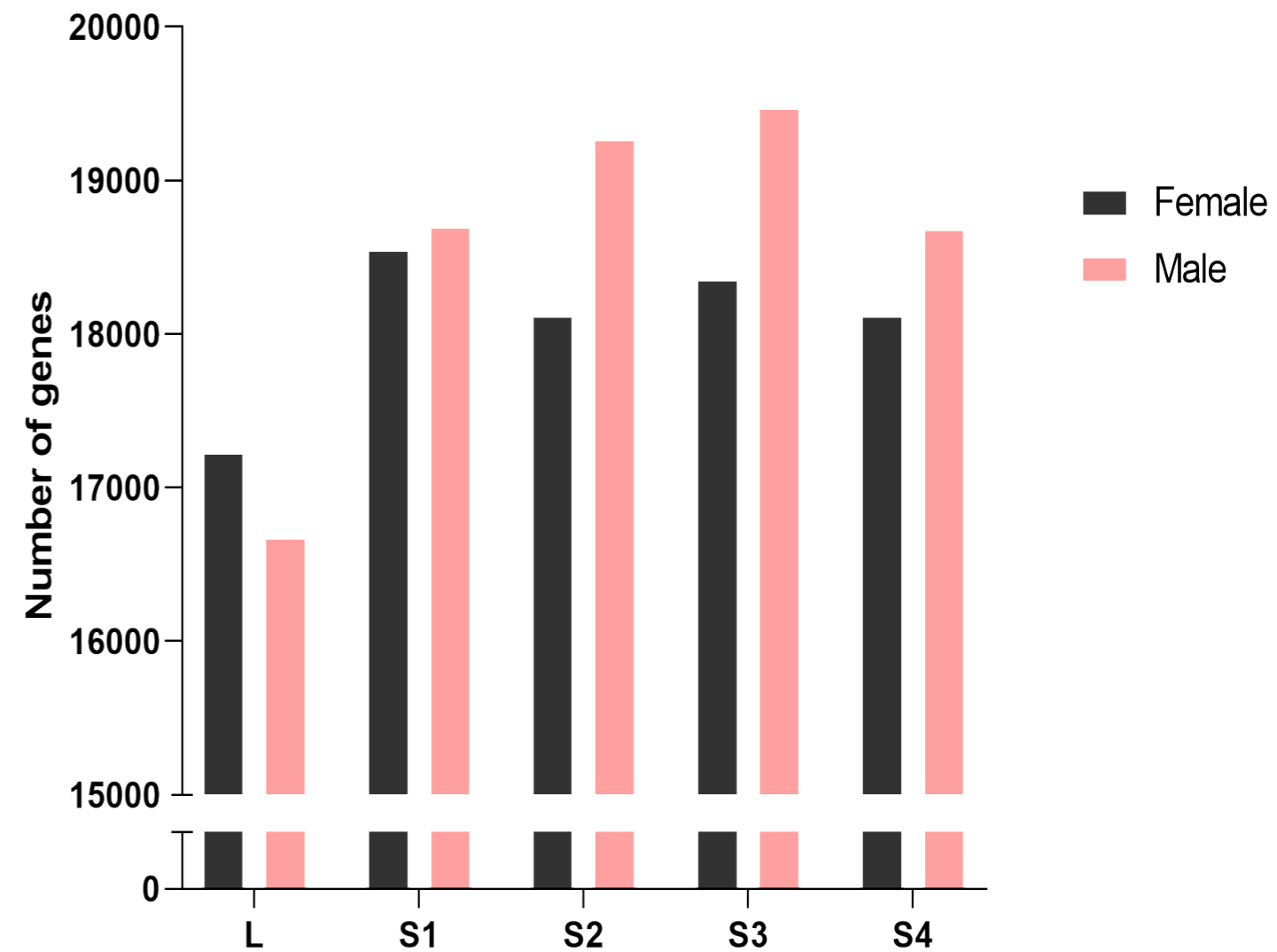

Supplement: Supplementary file 2 — Additional file 2. Global transcript analysis in different stages/samples. a, Count the expression ratio of high/medium/low expressed genes. Percentage is used to indicate the number of genes at different expression levels b, Comparison of the number of expressed genes between females and males. The height of the histogram represents the number of gene expressions in different stages. [file 12864_2021_8197_MOESM2_ESM.pdf]

a

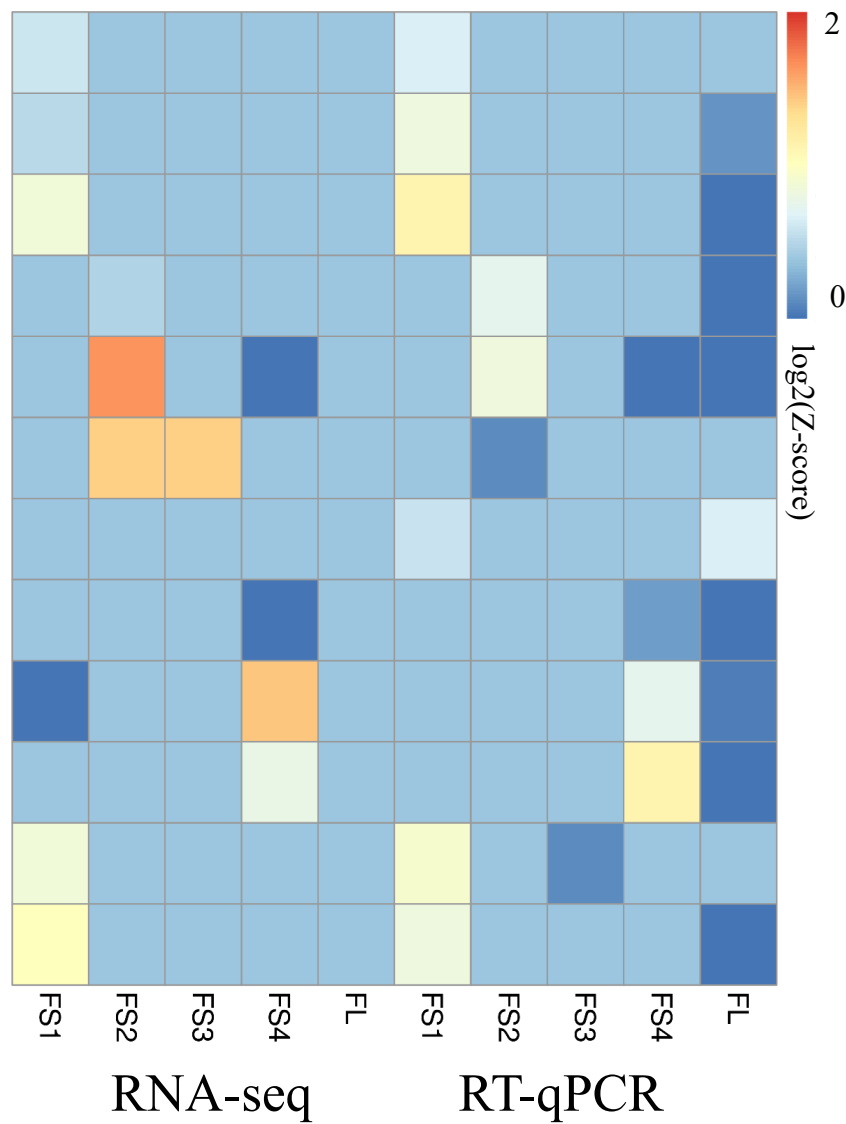

b

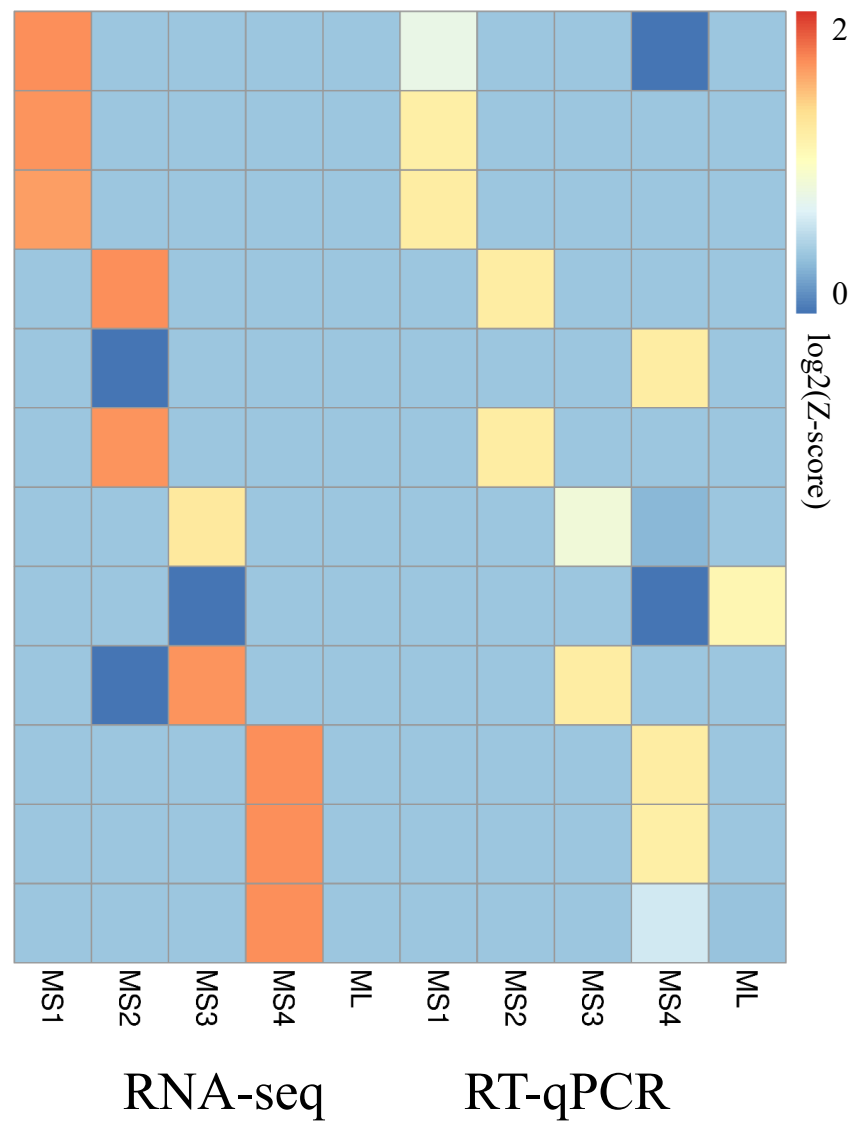

Supplement: Supplementary file 5 — Additional file 5. Comparison of gene expression in RT-qPCR experiment and RNA-seq data analysis. a, Female samples. b, Male samples. [file 12864_2021_8197_MOESM5_ESM.pdf]

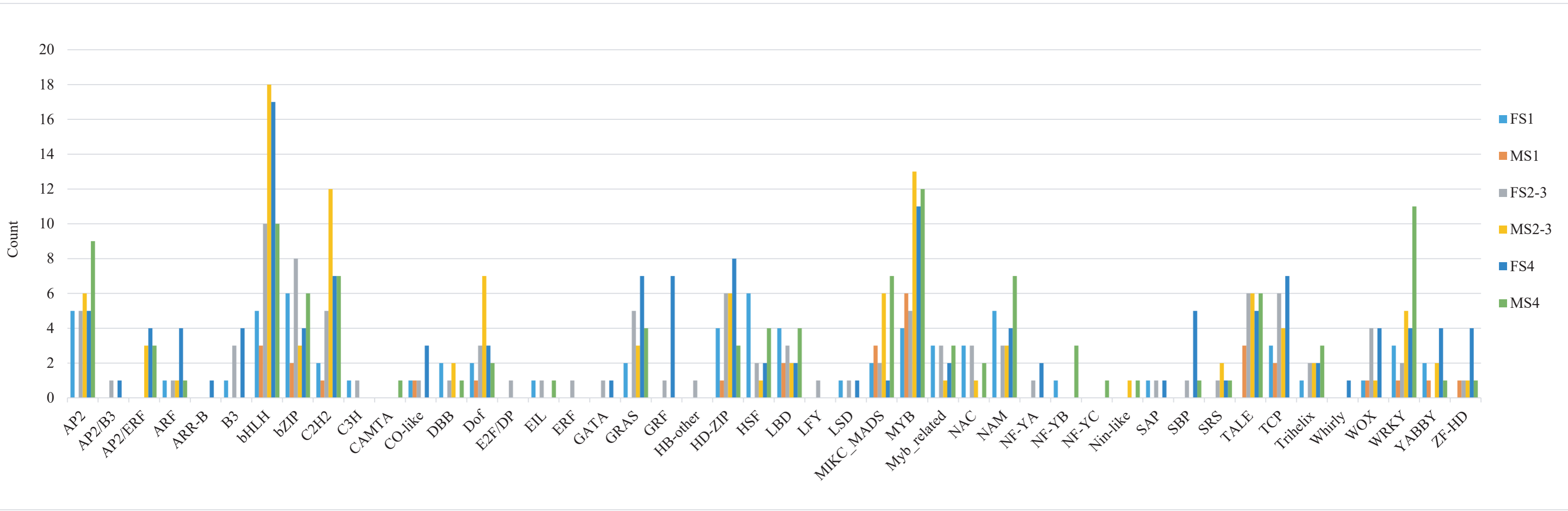

Supplement: Supplementary file 7 — Additional file 7. Bar chart of differentially expressed transcription factors between females and males at different stages. The abscissa shows the different transcription factor families. Different colors represent different stages of pistil development. [file 12864_2021_8197_MOESM7_ESM.pdf]

a

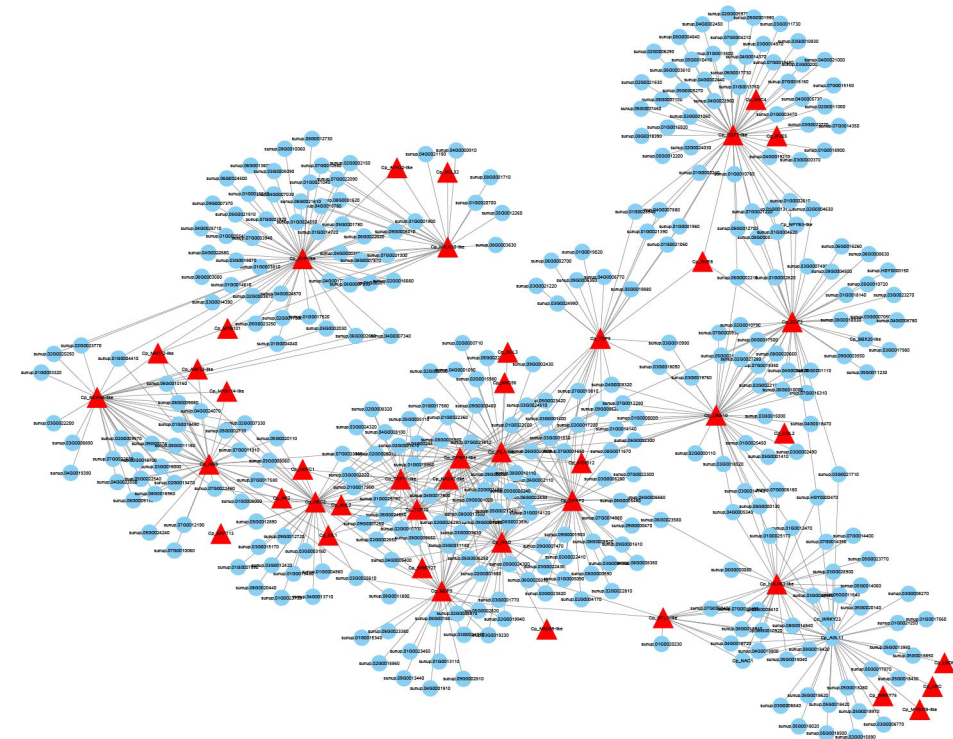

b

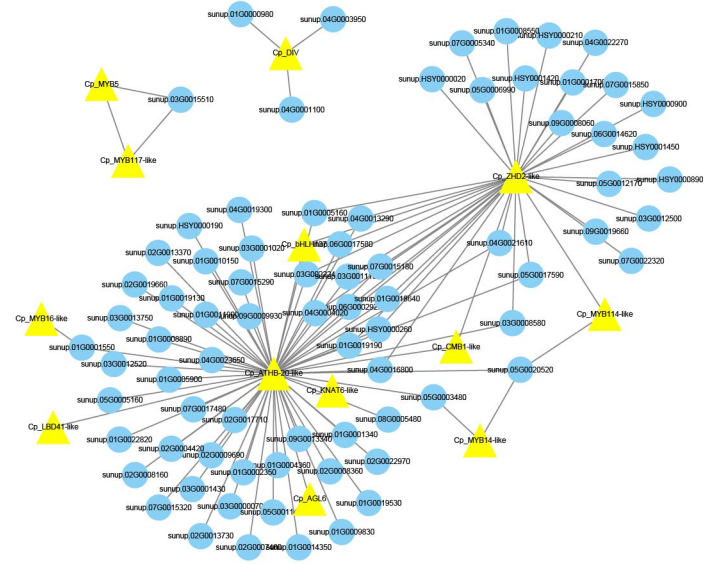

Supplement: Supplementary file 8 — Additional file 8. Diagram of gene regulation network in primordium stage (S1), red triangles represent transcription factors, blue circles represent other genes. The black lines link the regulatory relationships between genes a, Female sample. b, Male sample. [file 12864_2021_8197_MOESM8_ESM.pdf]

a

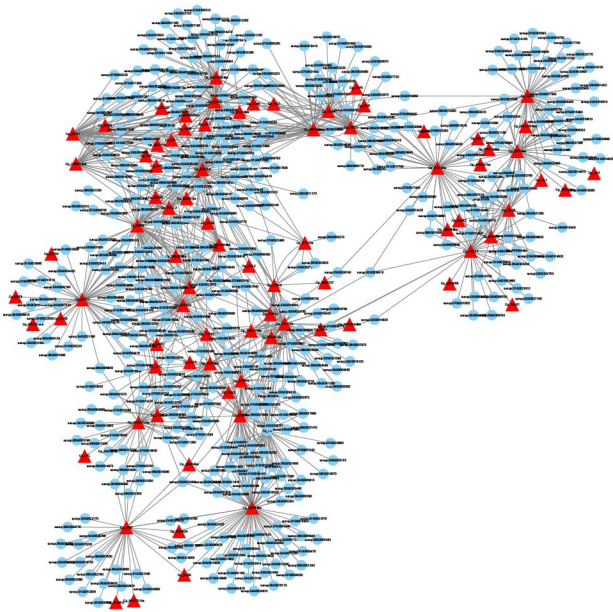

b

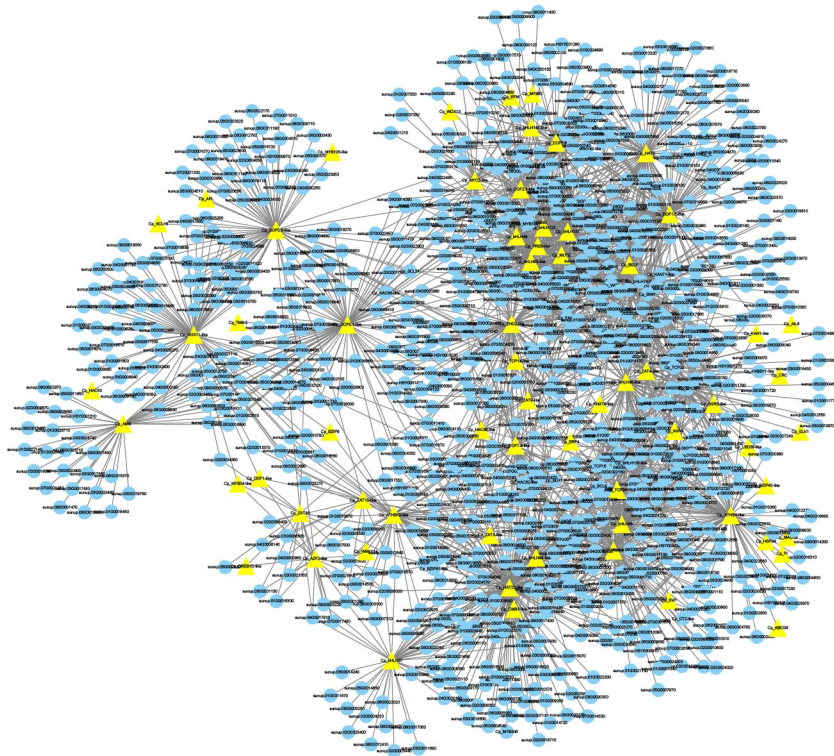

Supplement: Supplementary file 9 — Additional file 9. Diagram of gene regulation network in meiosis stage (S2-3), red triangles represent transcription factors, blue circles represent other genes. The black line represents the regulatory relationship. a, Female sample. b, Male sample. [file 12864_2021_8197_MOESM9_ESM.pdf]

a

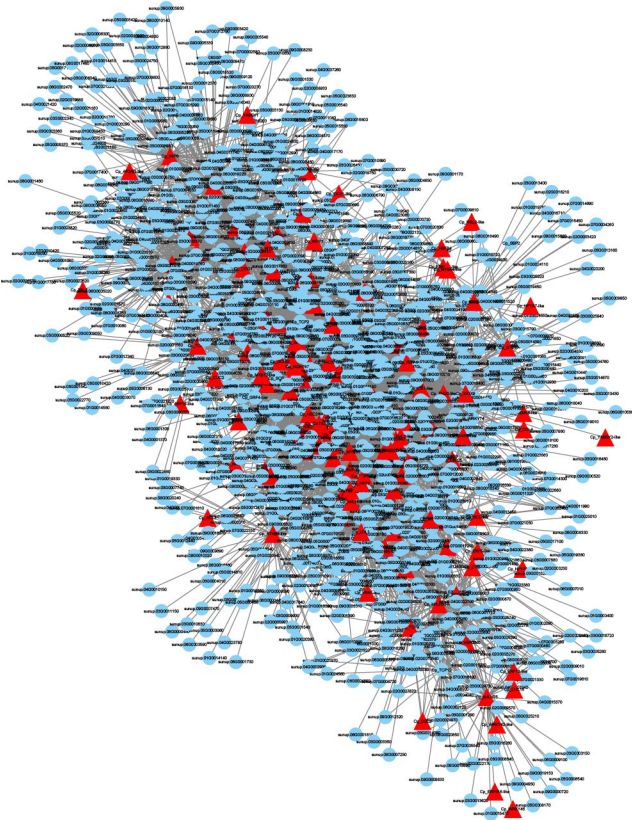

b

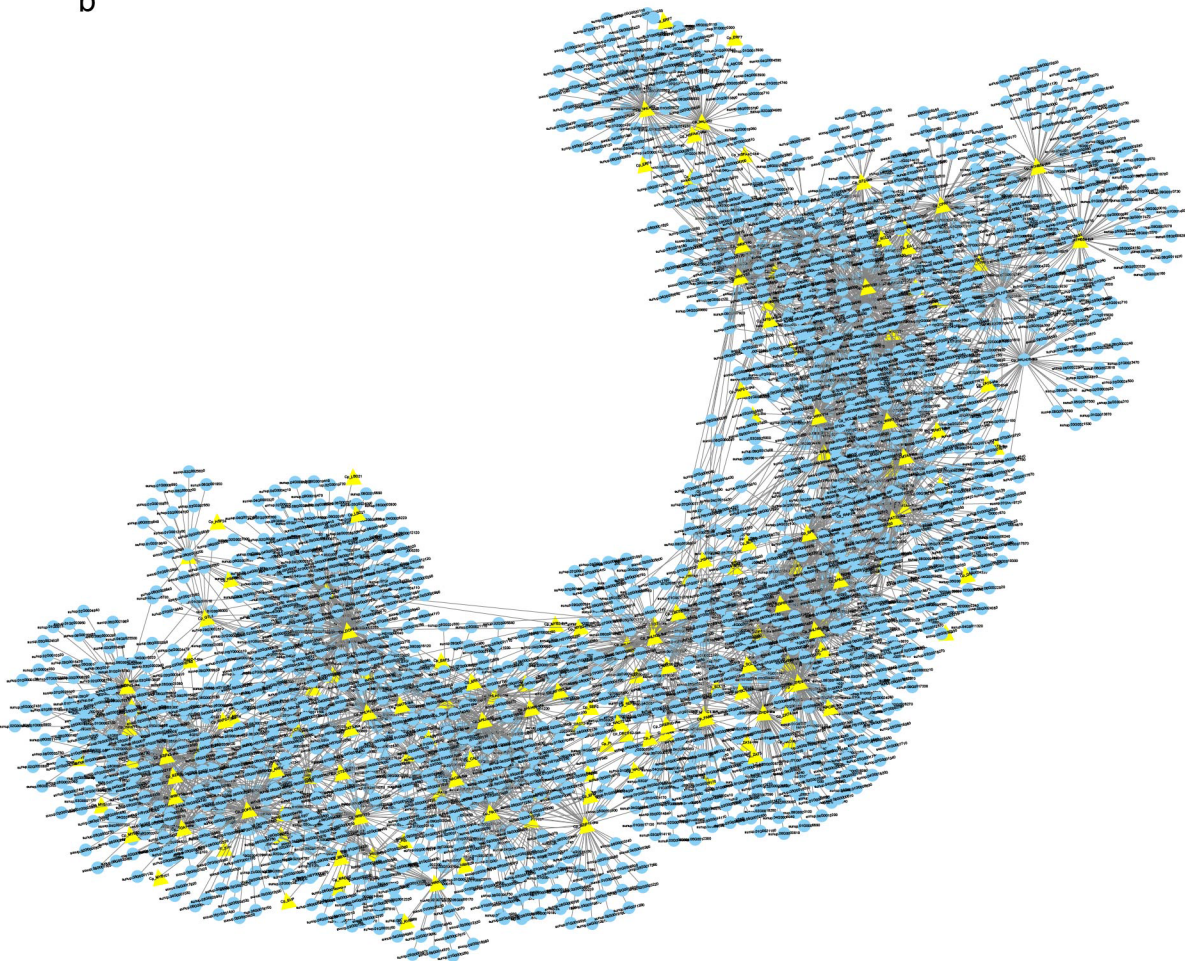

Supplement: Supplementary file 10 — Additional file 10. Diagram of gene regulation network in mitosis stage (S4), red triangles represent transcription factors, blue circles represent genes. Transcription factors and regulated genes are connected with black lines. a, Female sample. b, Male sample. [file 12864_2021_8197_MOESM10_ESM.pdf]

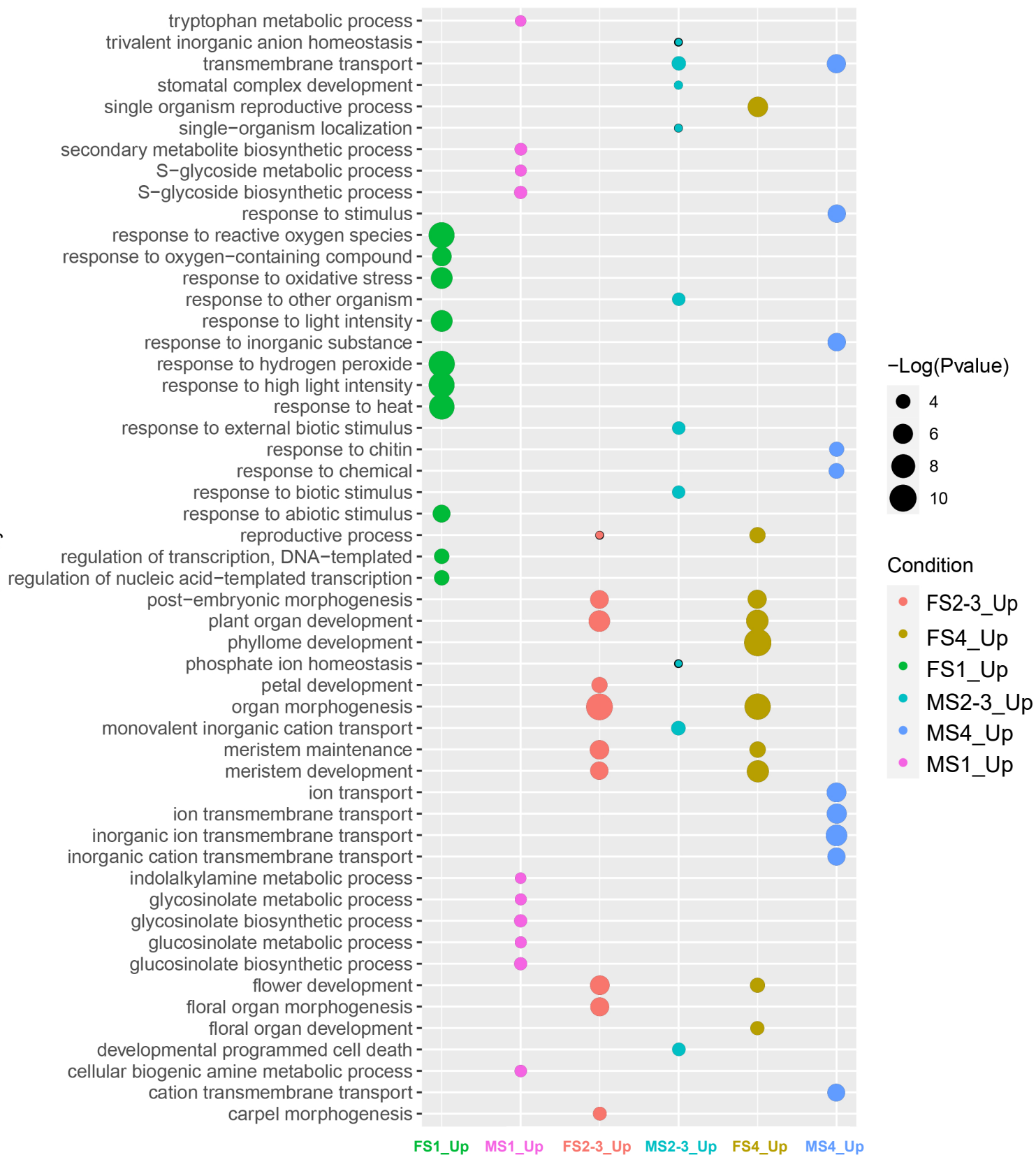

Supplement: Supplementary file 13 — Additional file 13. Bubble chart of GO enrichment analysis of gene regulatory network in different stages. Different bubbles visually show the sharing and unique pathways of different networks. [file 12864_2021_8197_MOESM13_ESM.pdf]

### *CpHEC2*

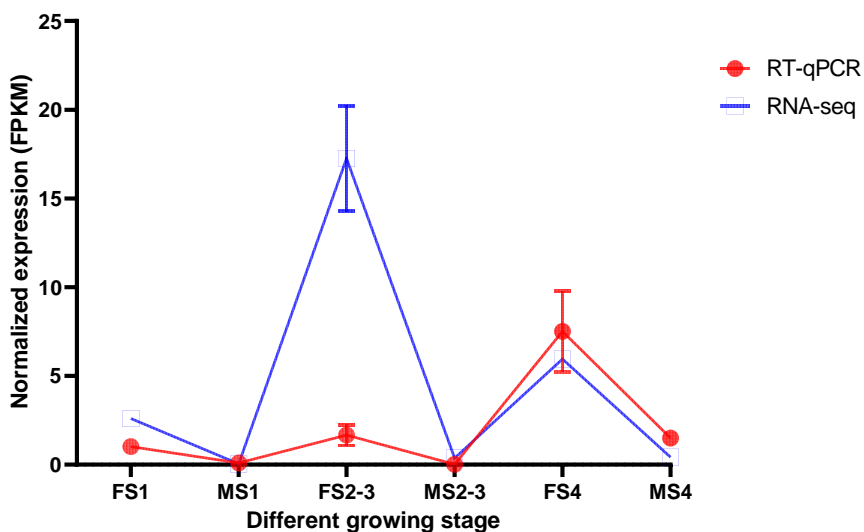

### *CpSUPL*

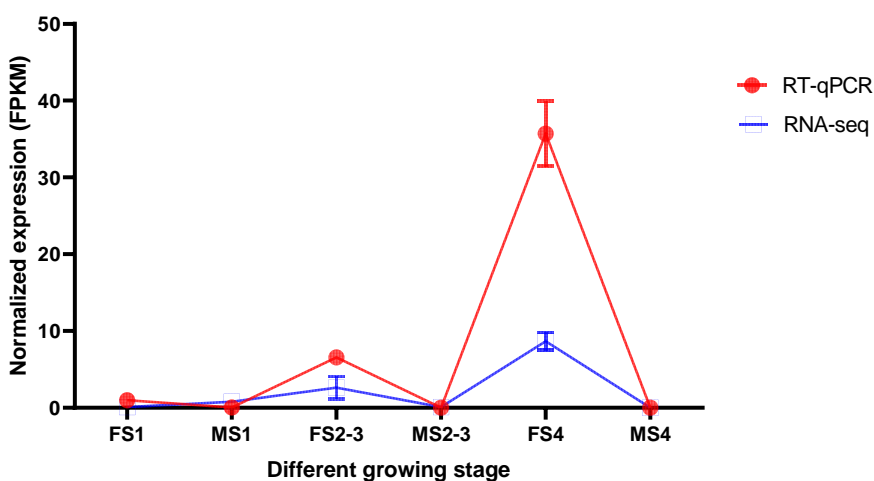

### *CpAGL11*

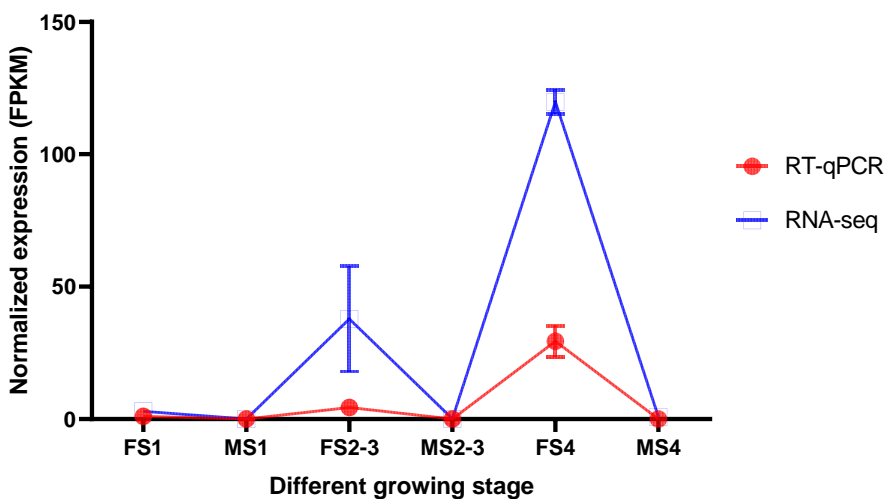

Supplement: Supplementary file 14 — Additional file 14. RT-qPCR to verify the expression pattern of CpHEC2, CpSUPL, and CpAGL11 genes. The Y coordinate represents the normalized expression, and the X coordinate represents different developmental stages. [file 12864_2021_8197_MOESM14_ESM.pdf]
